# Sex differences in peripheral not central immune responses to pain-inducing injury

**DOI:** 10.1038/s41598-017-16664-z

**Published:** 2017-11-28

**Authors:** Douglas M. Lopes, Natalia Malek, Michelle Edye, Sara Buskbjerg Jager, Sheridan McMurray, Stephen B. McMahon, Franziska Denk

**Affiliations:** 1 King’s College London, London, United Kingdom; 2 Laboratory of Pain Pharmacology, Institute of Pharmacology, Polish Academy of Sciences, Krakow, Poland; 3 Department of Biomedicine, Aarhus University, Aarhus, Denmark

## Abstract

Women suffer chronic pain more frequently than men. It is not clear whether this is due to differences in higher level cognitive processes or basic nociceptive responses. In this study we used a mouse model of neuropathic pain to dissociate these factors. We performed RNA-seq on purified peripheral afferent neurons, but found no striking differences in gene expression between male and female mice, neither before nor after nerve injury. Similarly, spinal cord immune responses between the sexes appeared to be indistinguishable when studied by flow cytometry or qRT-PCR. Differences emerged only upon studying peripheral immune cell infiltration into the dorsal root ganglion, suggesting that adaptive immune responses in neuropathic pain could be sexually dimorphic.

## Introduction

Women are more likely to suffer from common pain conditions, such as migraine, back pain or osteoarthritis^1^, and painful autoimmune disorders, like rheumatoid arthritis^2^. Despite clinicians encountering this sex difference on a daily basis, its biological underpinnings are still poorly understood. Women might simply be more prone to pain-generating injury or disease, though laboratory experiments also suggest the presence of a sex difference in pain sensitivity *per se* ^1,3,4^.

Two broad categories of mechanism have been proposed for this difference in pain sensitivity. There are those that implicate complex cortical processes which can modulate pain perception, such as anxiety or gender-specific motivations. Alternatively, it has been suggested that women process noxious stimuli differently at the most basic level, *i.e*. in their peripheral nerves or spinal cord. Evidence for the latter derives mostly from the pre-clinical literature, where studies in knockout mice have implicated various nociceptive mediators in sex-specific pain and analgesia responses^1^. More recently, there have also been several reports of fundamental differences in spinal cord neuroimmune interactions in male and female mice^5–7^.

Yet, most studies to date have relied on behavioural readouts and lack more detailed examinations of cellular and molecular mechanisms. Here, we characterised sex differences in peripheral neurons and immune cell populations to further address the question of whether the origin of sexual dimorphisms in pain should be sought at the level of basic nociception.

## Results

We first investigated whether primary afferent nociceptors – known to be essential drivers of chronic pain - have distinctive molecular profiles in male and female mice. RNA sequencing was performed on purified sensory neurons using either fluorescence-activated cell sorting (FACS) in naïve mice (n = 11, Supplementary Figure 1) or magnetically activated cell sorting (MACS)^8^ in mice that had undergone a pain-inducing nerve injury (n = 3, partial sciatic nerve ligation, SNL). In contrast to most other RNA-seq experiments, our FACS study had an unusually large number of replicates, affording us the power to detect a two-fold change with high certainty, even for transcripts with lower expression level (Supplementary Table 1). Despite this, we only identified 7 genes that were differentially expressed between male and female DRG neurons in a naïve state (Supplementary Tables 2 and 3). The majority of them were X- or Y-linked and have also been reported as differentially expressed in human^9^ and mouse brain^10^.

**Figure 1:**
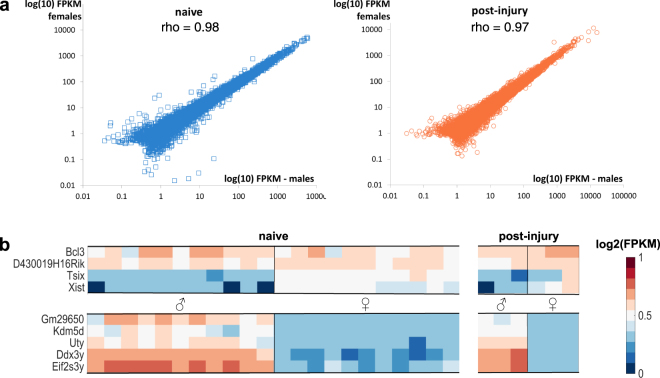
Transcriptionally, sensory neurons purified from adult male and female mice are very similar. (**a**) Plots of average FPKM values of male versus female RNA-seq samples on a log(10) scale. Sensory neurons were derived from naïve (n = 11) as well as post-injury DRG (SNL, day 8, n = 3). Spearman’s rank correlations (rho) are displayed on the graphs. (**b**) Genes found to be differentially expressed (DESeq2) at adj. p < 0.05 in naïve (n = 11, right) or post-injury (SNL, day 8, n = 3, left) male and female mice. Heatmaps display log2 (FPKM) values.

**Figure 2:**
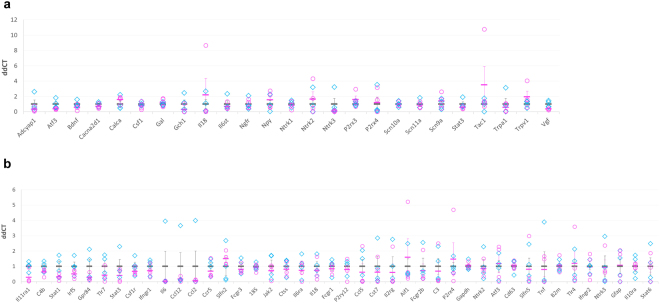
qRT-PCR of purified sensory neurons and spinal cord immune cells show no significantly dysregulated genes between male and female mice after nerve injury. (**a**) Nociceptive neurons were isolated from ipsilateral L3-L5 lumbar DRG of mice using magnetically activated cell sorting and qRT-PCR performed on the resulting RNA. No significant differences emerged between male and female mice after multiple comparison corrections (p > 0.05, n = 4). Displayed are individual ddCT values for each biological replicate (blue for males, pink for females), as well as group means and standard errors (grey for males, pink for females). (**b**) Immune cells (98% microglia) were isolated with a Percoll gradient from ipsilateral lumbar spinal cord eight days after SNL. A panel of 39 genes related to microglial activation, nociception and female adaptive immunity were tested for their expression levels using qRT-PCR. No significant differences emerged between male and female mice after multiple comparison corrections (p > 0.05, n = 4). Displayed are individual ddCT values for each biological replicate (blue for males, pink for females), as well as group means and standard errors (grey for males, pink for females).

The same was true after SNL, where a very similar set of 7 genes was identified (Fig. 1, Supplementary Tables 3 and 4). For this sequencing experiment, we had a more usual n of three per group and therefore chose to perform a qRT-PCR validation experiment in an independent cohort of mice (n = 4). We chose 25 well-established nociception-related genes, but again found expression levels to be indistinguishable between the sexes (Fig. 2a, Supplementary Table 5). In our datasets therefore, we could not identify any transcriptional differences in the peripheral nociceptive neurons of male and female mice.

**Figure 3:**
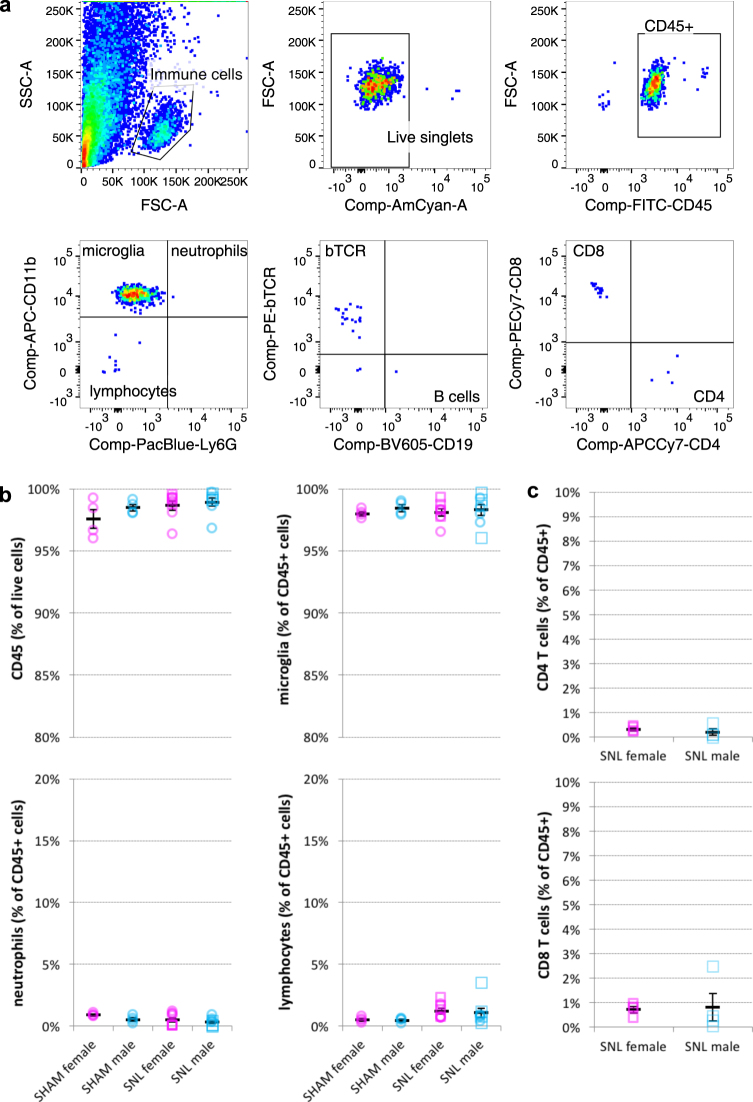
Flow cytometry analysis of mouse spinal cord after SNL reveals very few immune cells besides microglia and no obvious sex differences. (**a**) Gating strategy of Percoll-isolated immune cells from ipsilateral lumbar spinal cord. Live single immune cells were selected and identified based on CD45+. The numbers of CD11b+ microglia, CD11b+/Ly6G+ neutrophils and CD11b−/Ly6G− lymphocytes were compared across male and female mice. Lymphocytes were further classified into CD19+ B cells and βTCR+ T cells (bTCR), which were split according to their CD4 and CD8 positivity (**b**,**c**). Quantification of flow cytometry data obtained eight days after SNL or sham surgery. Plotted is the percentage of CD45+ amongst all live cells and the percentage of microglia, neutrophils, lymphocytes, CD8+ and CD4+ T cells amongst CD45+ immune cells. Each dot represents a mouse (females in pink, males in blue) from two independent experiments displayed as circles (n = 4) and squares (n = 4). Black bars are means + SE. No significant differences were found between male and female mice.

To investigate whether some of the reported sex differences might arise at the level of the immune response, as has previously been suggested^5^, we also examined peripheral and spinal cord immune cells. As we and others reported before^11,12^, we could identify very few immune cells besides microglia in the spinal cord eight days after SNL (Fig. 3) – the time at which spinal T cell involvement in sex differences has been hypothesised^5^. The same was true 24 days after SNL (Supplementary Figure 2).

Moreover, there were no differences in immune cell numbers between male and female mice in the spinal cord: microglial proliferation rates were comparable (Supplementary Figure 3) and different immune cell types were observed at similar frequencies (Fig. 3). Finally, no sex specific differences in gene expression could be detected either in naïve mice or after SNL; we tested select panels of genes known to be involved in microglial activation, nociception or female adaptive immunity, including P2rx4, Tlr4, and Tlr7 (Fig. 2b, Supplementary Figure 4 and for raw data see Supplementary Tables 6 and 7).

**Figure 4:**
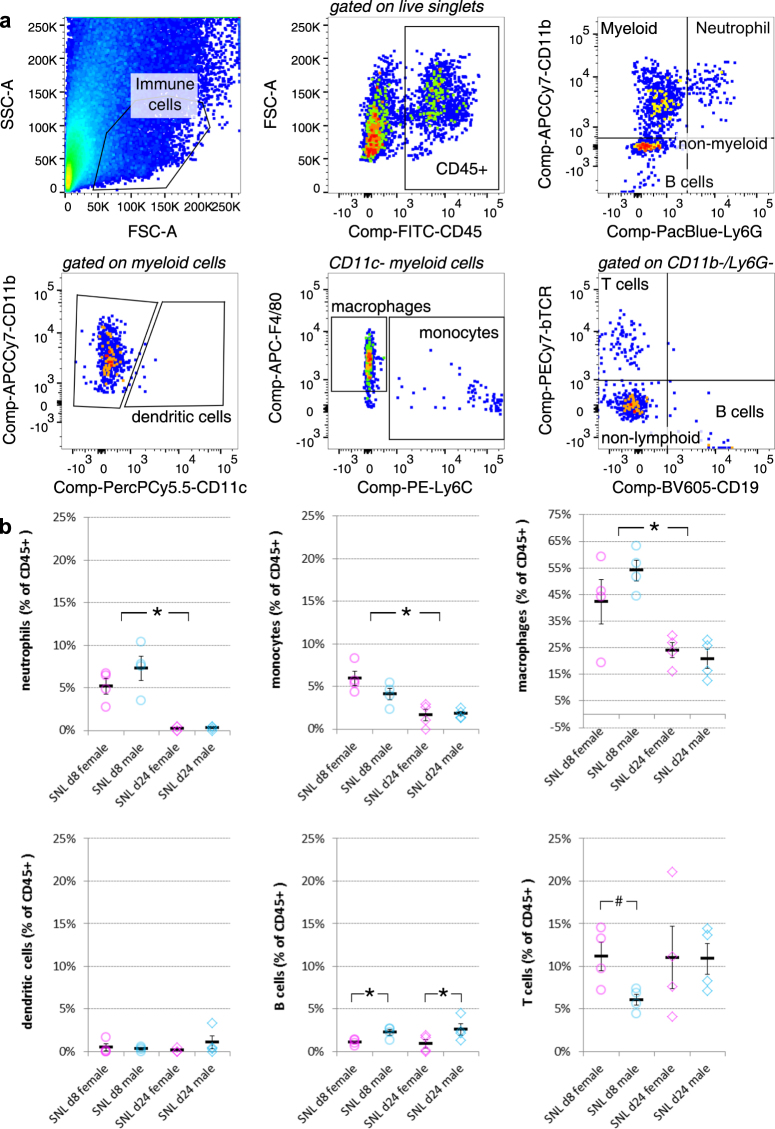
Flow cytometry analysis of peripheral DRG after SNL reveals potential sex differences in adaptive immune cell types. (**a**) Gating strategy of ipsilateral L3-L5 DRG of male and female mice. Live single immune cells were selected and identified based on CD45+. Ly6G and CD11b were used to distinguish neutrophils and cells of the myeloid lineage. Myeloid cells were further split into CD11c+ dendritic cells, F4/80+ macrophages and Ly6C+ monocytes. Lymphoid cells were subdivided into βTCR+ T cells and CD19+ B cells. There was also a substantial CD11b−, Ly6G− population that did not appear to be of lymphoid lineage. (**b**) Quantification of flow cytometry data obtained eight days or 24 days after SNL. Plotted is the percentage of neutrophils, monocytes, macrophages, dendritic cells, B cells and T cells amongst live CD45+ immune cells. Each dot represents a mouse (females in pink, males in blue) from two independent experiments displayed as circles (n = 4) and diamonds (n = 4). Black bars are means + SE. *p < 0.012, ^#^p < 0.05.

In contrast to the spinal cord, we detected significant immune cell infiltration into the DRG upon injury, as expected. More neutrophils, monocytes and macrophages were identified eight days after SNL compared to 24 days (two-way ANOVA, *main effect of time*, p < 0.001, Fig. 4, Supplementary Figure 5). More interestingly, we found hints that the response of adaptive immune cells might be different in male and female mice. That is, a significantly increased frequency in B cells was detected in male DRG eight days after SNL (two-way ANOVA, *main effect of sex*, p = 0.011), and T cells appeared more frequent in female mice (independent samples t-test, p = 0.048).

## Discussion

Our data did not support a sex difference in basic nociceptive neuron biology, at least not at the transcriptional level. In naïve neurons, we can be confident of this finding, as we were unusually well powered to detect changes even in lowly expressed transcripts. After injury, we used a more conventional n number for RNA-seq (n = 3) and had a 70% chance to detect a two-fold change in about half of all genes. More than 500 genes have been reported to be regulated by more than two-fold as a result of nerve injury in purified neurons^13^, so we are unlikely to have missed big differences, especially in our select group of nociception-related genes which we independently validated. However, it cannot be ruled out that more subtle sex differences are present in more lowly expressed transcripts. In this context, it is also worth contemplating that our power analyses indicated that conventional RNA-seq experiments using n = 2–4 will frequently be underpowered (see an illustrative example using real data in Supplementary Table 1).

We also found no evidence for differences in spinal cord immune responses. We are confident that the vast majority of immune cells in the spinal cord after injury are microglia. Moreover, our data indicate that their numbers and expression profiles do not differ drastically between the sexes, at least when considered at population level in ipsilateral segments. Very recent work on microglia suggests that they might display extremely localised responses^14,15^. It is a fascinating concept, which perhaps means that further exploration of potential sex differences in microglial responses to nociception would have to be carried out at a single cell or clonal level.

Finally, our data indicate a possible sex-specific response of peripheral immune cells to neuropathic pain. This novel finding fits well into the wider immunological literature^2,16^, where evidence for heightened adaptive immune responses in women is wide-ranging and well-established^2,16^.

In conclusion, based on our results and the wider literature, we would currently advocate that mechanistic explanations for the observed differences in pain perception between men and women be sought either at the level of the peripheral immune system or in more complex central nervous system processes.

## Methods

### Animals

AdvGFP mice and non-transgenic littermates were used for FACS sorting of DRG neurons (gensat.org: STOCK Tg(Avil-EGFP)QD84Gsat/Mmucd). Mice were bred in house (12 hour light-dark cycle) and used between 3 and 6 months of age. All other experiments were performed on C57Bl/6J mice (age-matched, between 6 weeks and 7 months of age). All experiments described were carried out in accordance with the United Kingdom Home Office Legislation (Scientific Procedures Act 1986) and were approved by the Home Office to be carried out at King’s College London under project licence 70/0793.

### Surgery

Partial sciatic nerve ligation (SNL) was performed under isofluorane anaesthesia after injection of a single dose of 0.05 mg/kg buprenorphine for post-surgical analgesia. The sciatic nerve was exposed with blunt dissection of the overlying muscle and tightly ligated with a 5.0 vicryl suture. The wound was closed with a surgical clip. For sham surgeries, the sciatic nerve was exposed but not ligated.

### Estrus cycle identification

The estrus cycle of female mice was assessed via vaginal lavage (20μl of sterile water) and subsequent examination of cells under a light microscope. Cells were then air-dried and stained with hematoxylin and eosin for more permanent records (see Supplementary Figure 6). Once the cycle of each individual mouse had been established, partial sciatic nerve ligations were performed on females in estrus. Cells were harvested eight days later, when the females were once again in estrus (usually confirmed by another vaginal lavage).

Estrus cycle identification was performed for the following experiments: RNA-seq of MACS-sorted DRG neurons after nerve injury (Fig. 1 and Supplementary Tables 3 and 4), qRT-PCR experiments on spinal cord microglia after injury (Fig. 2b, Supplementary Table 6), flow cytometry experiments on spinal cord microglia and peripheral immune cells (Figs 3, 4, Supplementary Figure 2).

### FACS-sorting of DRG neurons

AdvGFP mice were sacrificed with an overdose of pentobarbital, perfused with 10 ml of PBS, and trigeminal (n = 2) or lumbar DRG (L1-L5, bilaterally, n = 9) dissected. Male and female mice were processed in sex-matched batches, with two mice of each sex pooled into one biological replicate. For each experiment, cells from a non-transgenic control animal were used for compensation controls.

After dissection, the ganglia were incubated in 3 mg/ml dispase, 0.1% collagenase and 200 U/ml DNAase for 60 min, followed by gentle trituration in F12 medium. The resulting single cell suspension was stained with propidium iodide (PI) to identify dead cells.

Fluorescence-activated cell sorting (FACS) was performed using a BD FACS Aria II Cell Sorter at the NIHR BRC flow core facility at King’s College London. To isolate neurons, cells were gated on GFP+, PI− signal with the help of unstained and single-staining controls. Positive events were sorted directly into Qiagen RLT buffer and beta-mercaptoethanol and stored at −80 °C until the entirety of biological samples were collected. Note that this method will result in satellite glial cell contamination, with approximately 50% of neurons remaining attached to their satellite glial cells after purification as measured by immunofluorescent staining (Supplementary Figure 7).

### MACS-sorting of DRG neurons

Eight days after nerve injury or sham surgery, C57BL/6J mice were sacrificed with an overdose of pentobarbital and perfused with 10 ml of PBS. Lumbar DRG (L3-L5) ipsilateral to the nerve ligation or sham injury were dissected, and a single cell suspension created as described above (‘FACS-sorting of DRG neurons’). Two mice per sex (or 6 DRG) were pooled into a single biological replicate, in sex controlled batches (n = 2 per batch).

For magnetic sorting, we used a Miltenyi Neuronal Isolation kit (cat. #130-098-752). Briefly, triturated cells were filtered through a 70 micron mesh and incubated in primary antibody cocktail (diluted 1:5 in MACS buffer) for 5 min at 4 °C. This was followed by a wash in MACS buffer and incubation with magnetic anti-Biotin beads for 10 min at 4 °C. The resulting solution was then run through Miltenyi LS columns following manufacturer’s instructions. Purified cells were collected by centrifugation and resuspended in Qiagen RLT buffer and beta-mercaptoethanol and stored at −80 °C until all biological samples were collected. Note that this method, despite PBS perfusion, will result in some erythrocyte contamination and only isolates small diameter nociceptive C fibres. In contrast to FACS sorting, the neurons are however free of satellite glial cell contamination^8^.

### Microglial isolation

This protocol has been described previously^12,17^. Briefly, C57BL/6 J mice were sacrificed with an overdose of pentobarbital and perfused with 5 ml of PBS. Experiments were performed either eight days after nerve injury (Fig. 2b, Supplementary Figure 4B) or in a naïve state (Supplementary Figure 4A). Spinal cords were extracted using hydroextrusion and lumbar ipsilateral segments were isolated: the lumbar enlargement was identified visually, and the location of L5-L3 segments thus estimated and isolated using a fresh razor blade. Ipsilateral halves were obtained by dividing the segments in the middle of the dorsal horns, which are clearly visible as darker lines in the lighter, cream coloured cord. Mice were processed in sex matched batches. Once dissected, the tissue was dounce homogenized in mFACS buffer (HBSS with 0.4% BSA, 15 mM HEPES and 2 mM EDTA), spun down and resuspended in 70% Percoll, overlaid by a 37% Percoll solution. The gradient is spun for 30 min at 17 °C, after which cells are collected by two further centrifugation steps in HBSS and 0.2% BSA and resuspended in RNA lysis buffer (Qiagen RLT buffer and beta-mercaptoethanol) or mFACS buffer depending on the follow-up experiment.

### Peripheral immune cell extraction

Eight or 28 days after nerve injury, C57BL/6J mice were sacrificed with an overdose of pentobarbital and perfused with 10 ml of PBS. Ipsilateral lumbar DRG (L3-L5) were dissected into HBSS and incubated for 60 min at 37 C in 3 mg/ml dispase, 0.1% collagenase and 200 U/ml DNAase. The digestion solution was removed & replaced with 200 µl of mFACS buffer (see section on “Microglial isolation”). A single cell suspension of immune cells was then obtained by repeatedly triturating the ganglia with a P1000 pipette tip. Staining was carried out as described below.

### RNA extraction

Once all samples were collected for a given experiment, RNA extractions were performed in a single, sex-matched batch using a Qiagen RNeasy micokit, following manufacturer’s protocols with some minor modifications. RNA quality was assessed using an Agilent Bioanalyser PicoChiP (for RIN values of sequencing samples see Supplementary Table 8).

### Flow cytometry of spinal cord and peripheral immune cells

Immune cells were stained with Live/Dead fixable yellow dye (L-34959, Life Technologies) in HBSS for 15 min, followed by Fc block (purified anti-mouse CD16/32, Cambridge Bioscience, 101302, 1:20) and a cocktail of directly conjugated antibodies (1:300) from Biolegend for 15 min. A variety of different panels were used, e.g. CD45-FITC (103108), CD11b-APC (101212), Ly6G-PacBlue (127612), βTCR-PE (109208), CD19-BrilliantViolet605 (115540), CD4-APC/Cy7 (100526) and CD8a-PE/Cy7 (100722) for spinal cord immune cells or CD45-FITC (103108), CD11b-APC-Cy7 (101225), Ly6G-PacBlue (127612), CD11c-PercP/Cy5.5 (117328), Ly6C-PE (128007), F4/80-APC (123116), βTCR-PE/Cy7 (109222) and CD19- BrilliantViolet605 (115540) for peripheral immune cells. After fixation in 4% PFA for 5 min and resuspension in mFACS buffer, flow cytometry was performed on 200 μl of each cell solution using a BD SORP Fortessa at the NIHR BRC flow core facility at King’s College London. Unstained cells, cells stained with viability dye only and controls beads (BD Bioscience Comp beads, rat and hamster, cat. # 552845) stained with each antibody on its own were used for compensation. FlowJo was used to analyse the data. Basic gating for all events was as follows: all cells based on forward (FSC) and side scatter (SSC); single cells based on SSC area and width; live cells based on FSC and viability dye and finally FSC and CD45 for all immune cells. For quantification, all gates were kept constant between conditions.

### Proliferation assay

To assess microglial proliferation after nerve injury, we used a Click-iT™ EdU Alexa Fluor 488 Flow Cytometry Assay Kit (C10424, ThermoFisher Scientific). SNL or sham injured mice received daily 5-ethynyl-2′-deoxyuridine (EdU) injections (0.05 mg/ml injected in 400 μl *i.p*.) from the day of surgery until one day before their sacrifice on day 7. Mice were processed in sex and injury-matched batches: one SNL male, one SNL female, one sham male and one sham female per day. A completely naïve mouse and a naïve mouse that had also received daily EdU injections were used to generate unstained and FMO controls, respectively. Spinal cord immune cells were isolated with a Percoll gradient as described under “Microglial isolation” and labelled with CD45-PE, CD11b-BUV737 and Live/Dead fixable Near-IR dye (L-34976, Life Technologies) as above. Subsequently, the cells were fixed, permeabilized and stained for EdU according to the kit instructions. Flow cytometry data were collected and analysed as described above.

### qRT-PCR

After microglial extraction, RNA was amplified and converted to cDNA using a QIAGEN Repli-G single cell WTA kit according to manufacturer’s instructions. After quantification with a Qubit ssDNA assay kit, 6 ng/μl of cDNA were loaded onto Taqman microfluidic array cards or used in regular SYBR Green qRT-PCR reactions. The ddCT method was used for analysis, using various different housekeeping genes: *Gapdh*, *B2m* and *18s* in the case of spinal cord immune cells; *Gapdh*, *Ywhaz* and *18s* in the case of sensory neurons. See Supplementary Tables 5–7 for probe IDs, primer sequences, data and analyses.

### Sequencing

RNA was sent for batch-controlled library preparation (SMARTer Ultra Low Input HV kit, 634820, Clontech) and sequencing at the High-Throughput Genomics Group at the Wellcome Trust Centre for Human Genetics at Oxford University. After amplification, samples were multiplexed in replicate flow cells on an Illumina HiSeq4000 platform to yield 75 bp reads of at least 25M read depth.

For analysis, fastq files were aligned to the mouse genome (mm10 assembly) using STAR (with–outFilterMultimapNmax 1)^18^. The resulting bam files were merged across lanes if necessary using samtools. A median of 85.6% reads could be uniquely mapped (see Supplementary Table 8 for alignment percentages for each sample). Expression level (fragments per kilobase of transcript per million mapped reads, FPKM) was determined using Cufflinks^19^ run on the Galaxy Freiburg server (including fragment bias, length and multi-read corrections). FPKM>= 1 was deemed to be an appropriate expression threshold for both FACS and MACS sorted datasets. For differential expression analysis, reads were counted using htseq^20^ on the Galaxy Freiburg server (mode: union), and DESeq2^21^ was run on R using standard parameters.

Sequencing data and supplementary files are accessible on GEO (GSE100035).

## Electronic supplementary material

Supplementary Information

Supplementary Dataset 1

Supplementary Dataset 2

Supplementary Dataset 3

Supplementary Dataset 4

Supplementary Dataset 5

Supplementary Dataset 6

Supplementary Dataset 7

Supplementary Dataset 8
