# Supplementary material for: Sex differences in peripheral not central immune responses to pain-inducing injury
Source: Sci Rep. 2017 Nov 28;7:16460. doi: 10.1038/s41598-017-16664-z (PMC5705679; doi:10.1038/s41598-017-16664-z)
Supplement: Supplementary file 1 — Supplementary Information [file 41598_2017_16664_MOESM1_ESM.pdf]

Supplementary Information

Sex differences in peripheral not central immune responses to pain-inducing injury

Douglas M. Lopes[1], Natalia Malek[1, 2*], Michelle Edye[1], Sara Buskbjerg Jager[3], Sheridan McMurray[1], Stephen B. McMahon[1], Franziska Denk[1]*

[1] King's College London, London, United Kingdom; [2] Polish Academy of Sciences, Krakow, Poland; [3]Department of Biomedicine, Aarhus University, Aarhus, Denmark. [*] these authors have contributed equally to this work.

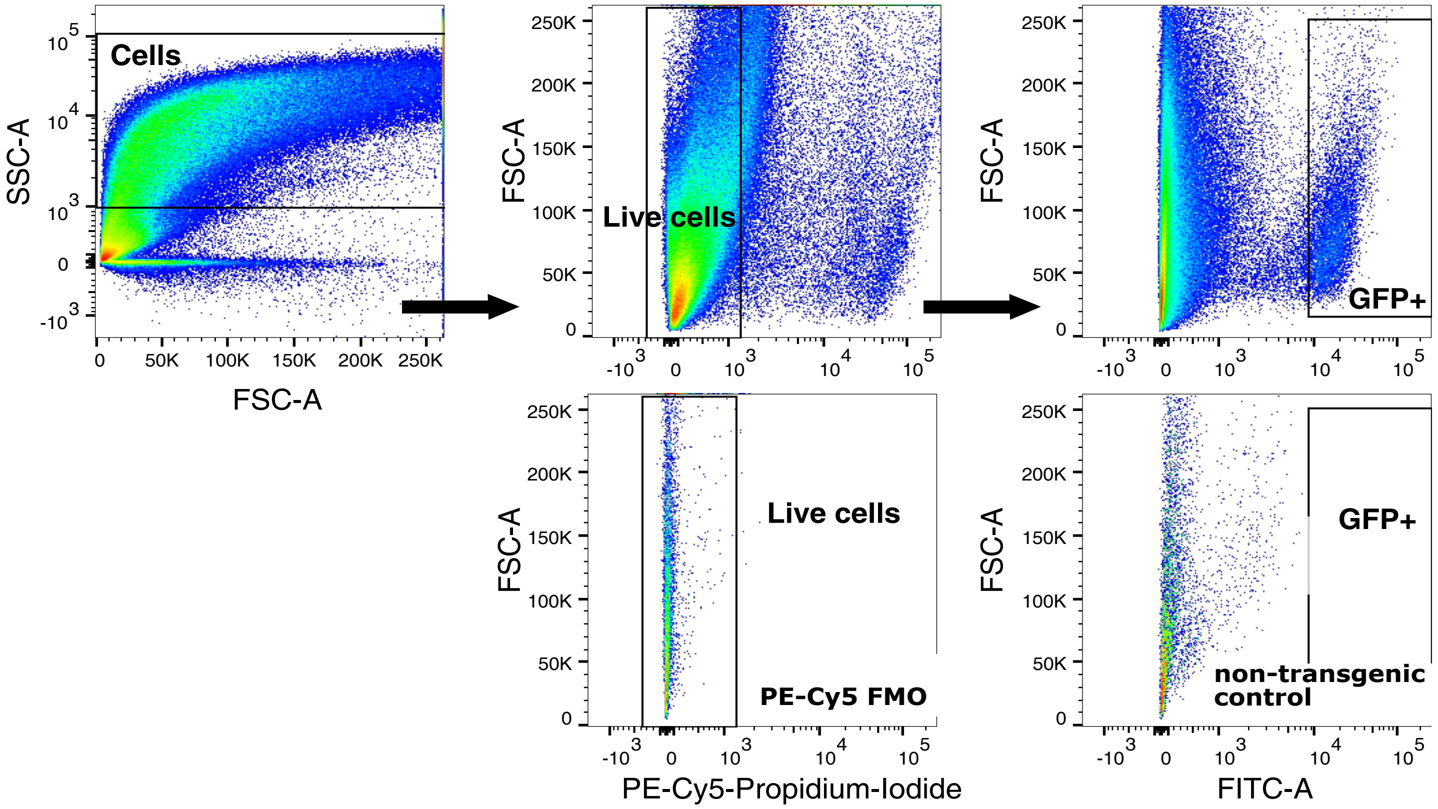

Suppl. Figure 1: Gating strategy for FACS of sensory neurons.
Neurons from AdvGFP positive mice were isolated using FACS by gating on live cells using propidium iodide (PI) and GFP positive cells. Cells from a non-transgenic littermate were used as an unstained control, and omission of PI was included as a fluorescence minus one (FMO) control for the live/dead stain.
Mice were processed in gender-matched batches, and lumbar DRG from two mice were pooled into each n for a total of 11 biological replicates.

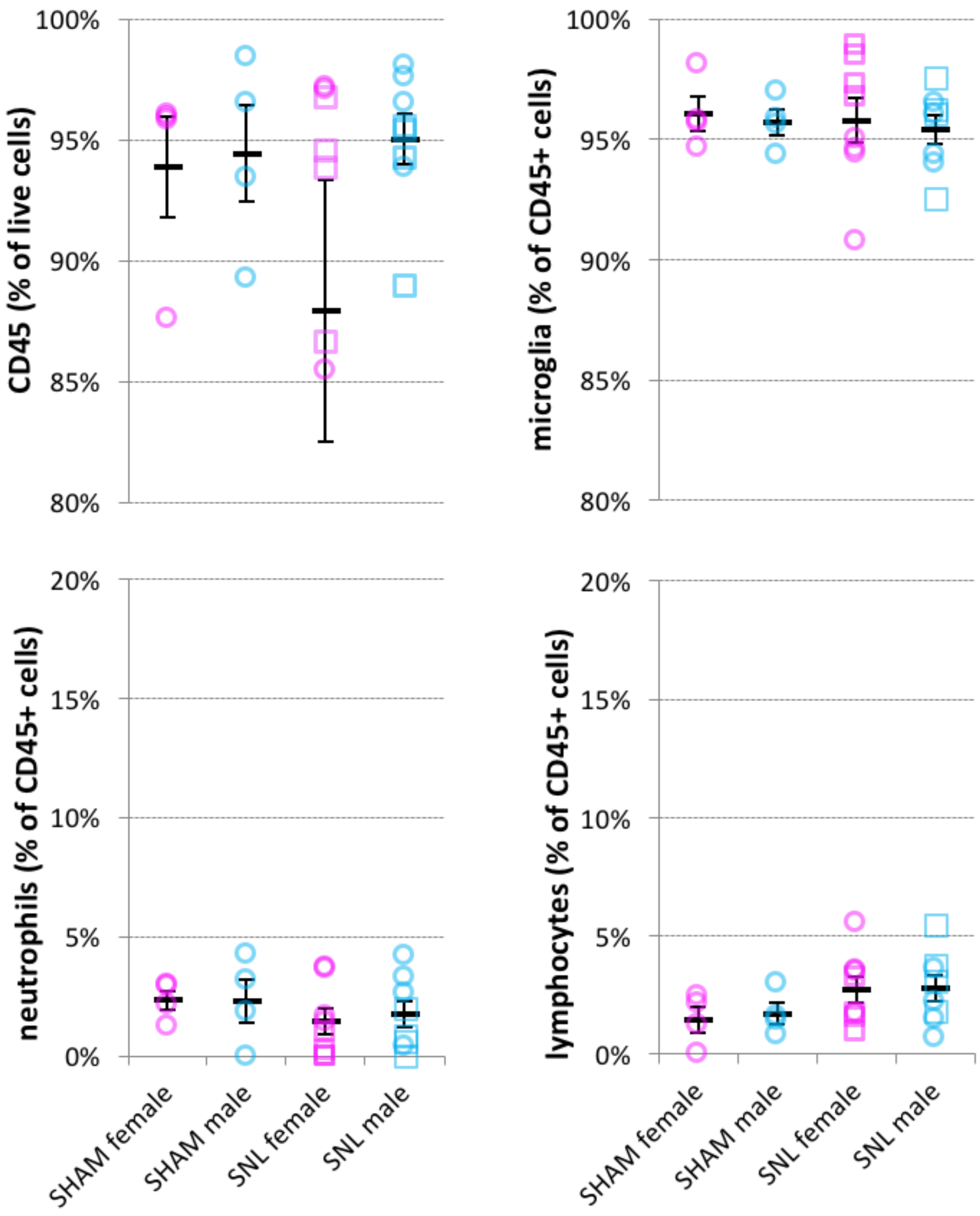

Suppl. Figure 2: Flow cytometry analysis of mouse spinal cord 24 days after SNL reveals very few immune cells besides microglia and no obvious sex differences. Quantification of flow cytometry data obtained 24 days after SNL or sham surgery. Plotted is the percentage of CD45+ amongst all live cells and the percentage of microglia, neutrophils and lymphocytes amongst CD45+ immune cells. Each pink dot represents a female mouse, while each blue dot represents a male mouse from two independent experiments displayed as circles (n = 4) and squares (n = 4). Black bars are means + SE. No significant differences were found between male and female mice.

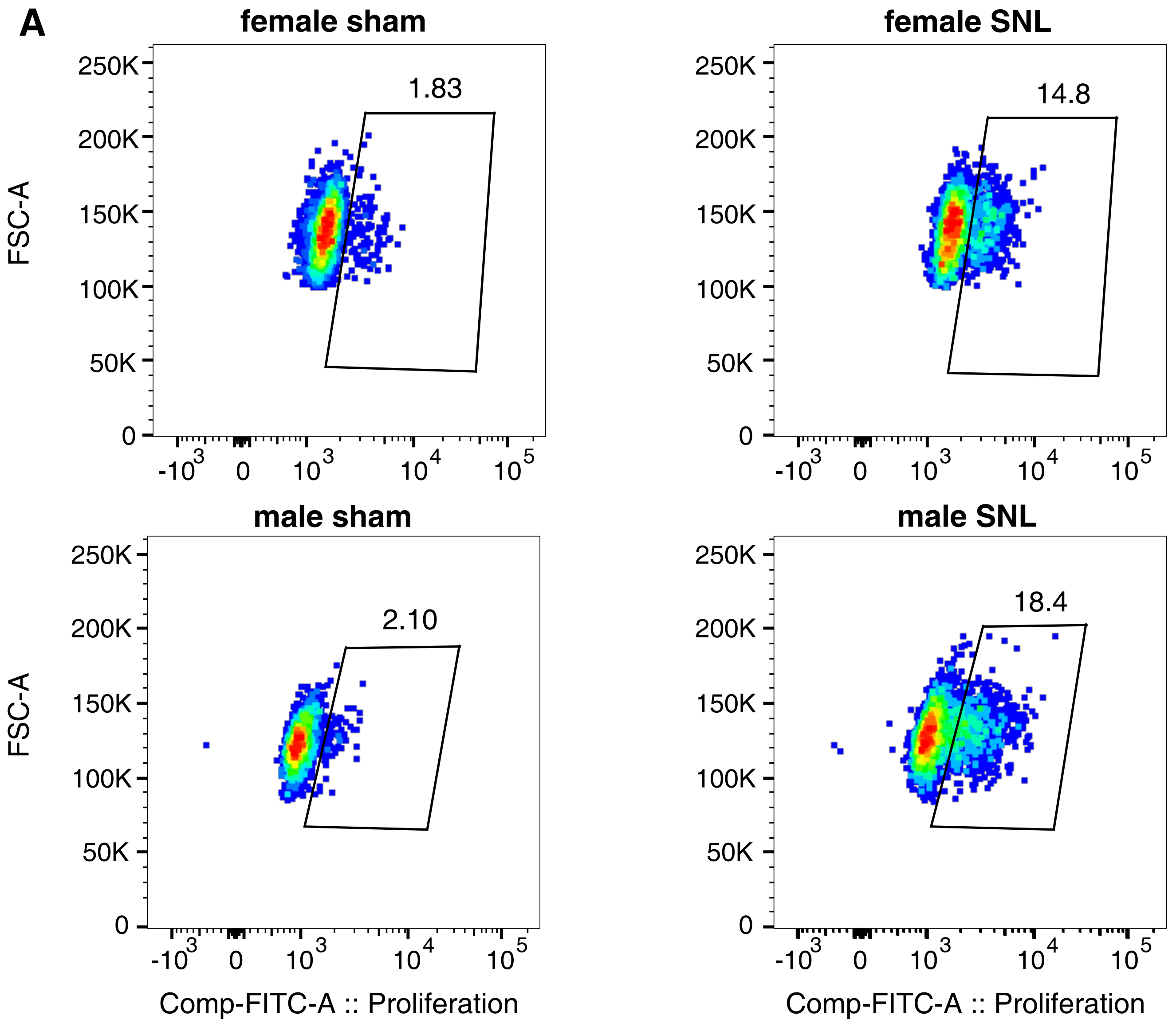

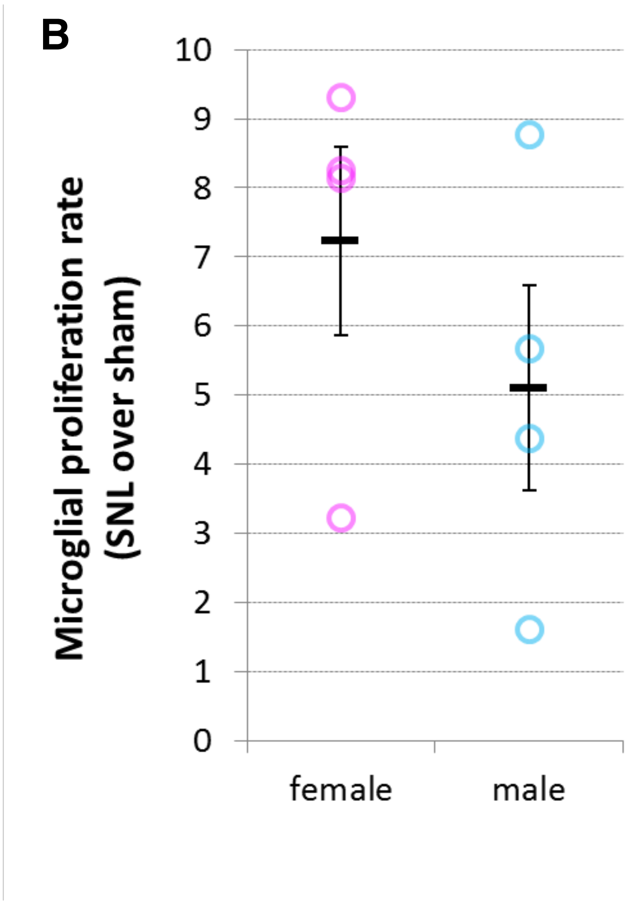

Suppl. Figure 3: Flow cytometry analysis of mouse spinal cord 7 days after SNL reveals an equivalent degree of microglial proliferation in male and female mice.

(A) Representative flow cytometry plots of Percoll isolated spinal cord microglia gated on CD45+/ CD11b++ live singlets. Significant proliferation, as measured by Edu incorporation, could be observed after nerve injury.

(B) Quantification of flow cytometry data obtained 7 days after SNL or sham surgery. Plotted is the ratio of proliferation comparing SNL over sham animals. Each dot represents a replicate pair (i.e. one SNL mouse vs its sham control, n = 4). Black bars are means + SE. No significant differences were found between male and female mice.

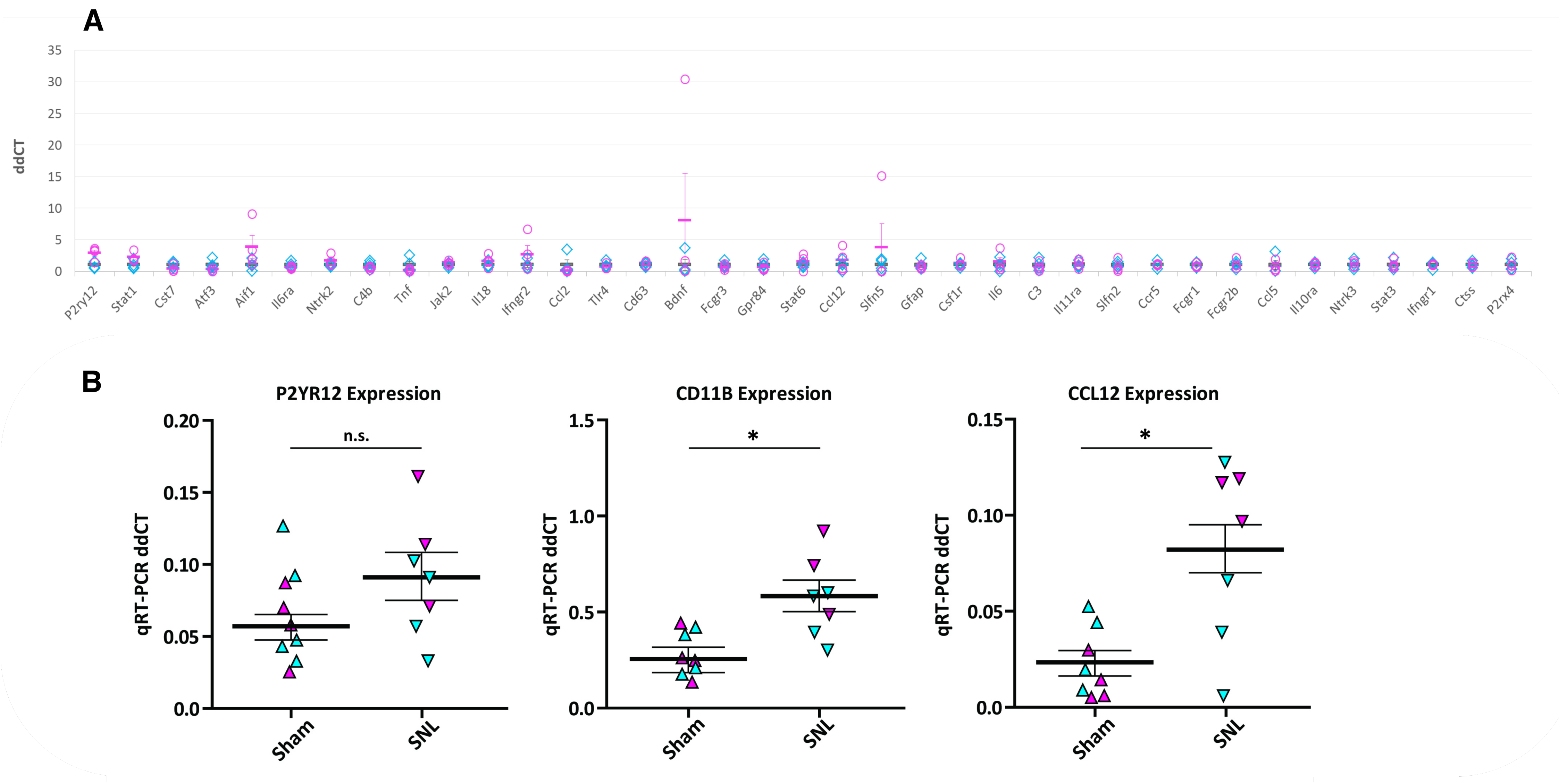

Suppl. Figure 4: qRT-PCR reveals no sex differences in naive microglia (A) and equivalent regulation of microglial genes after injury (B)

(A) Immune cells (98% microglia) were isolated with a Percoll gradient from the ipsilateral lumbar spinal cord of naive mice. A panel of genes related to microglial activation and nociception were tested for their expression levels using qRT-PCR. No significant differences emerged between male and female mice after multiple comparison corrections ($p < 0.05$, $n = 4$). Displayed are individual ddCT values for each biological replicate (blue for males, pink for females), as well as group means and standard errors (grey for males, pink for females).

(B) After SNL, both male and female mice showed equivalent levels of gene regulation, as measured by a few exemplary genes. Thus, as expected from RNA-seq data (12,15), Ccl12 and Cd11b mRNA was upregulated, while mP2RY12 RNA was unchanged. ANOVAs with *sex* and *injury* as independent variables revealed a significant difference between sham and SNL groups, but not male and female mice: main effect of *injury* (Ccl12: $p = 0.034$, Cd11b: $p = 0.049$), but no main effect of *sex* and no interaction between *sex* and *injury*.

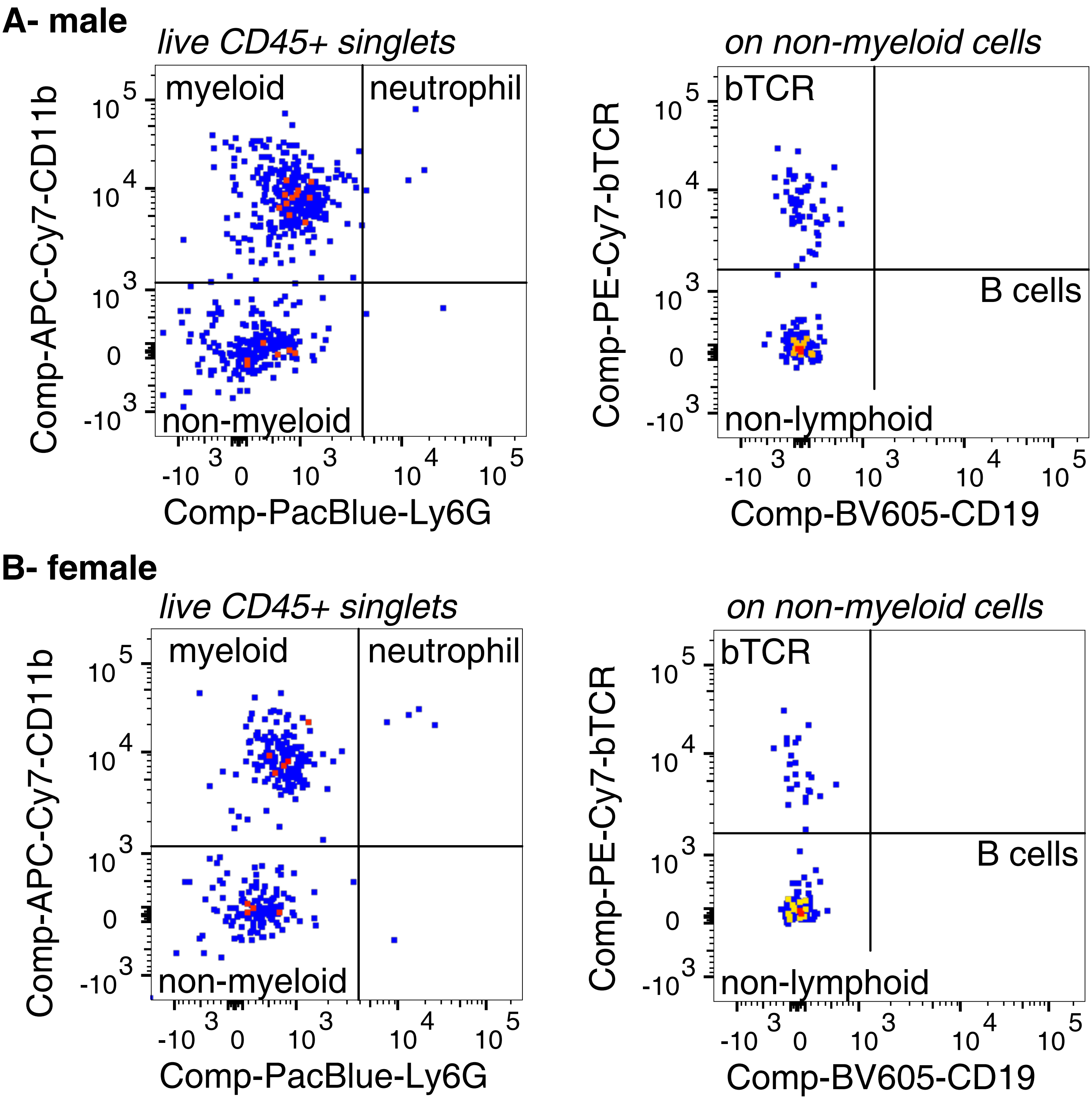

Suppl. Figure 5: Main immune cell populations identified in naive DRG
Three main immune cell populations were identified in naive mouse DRG via flow cytometry: myeloid cells (95%-98% of which were macrophages), βTCR-positive T cells and a non-lymphoid population - possibly consisting of CD11b and CD11c negative dendritic cells. We observed no CD11C+ dendritic cells or B cells, and minimal numbers of monocytes and neutrophils. There was no obvious difference in immune cell number between male and female DRG. Shown here are representative flow cytometry plots of data derived from three separate DRG pools obtained from one male and one female mouse.

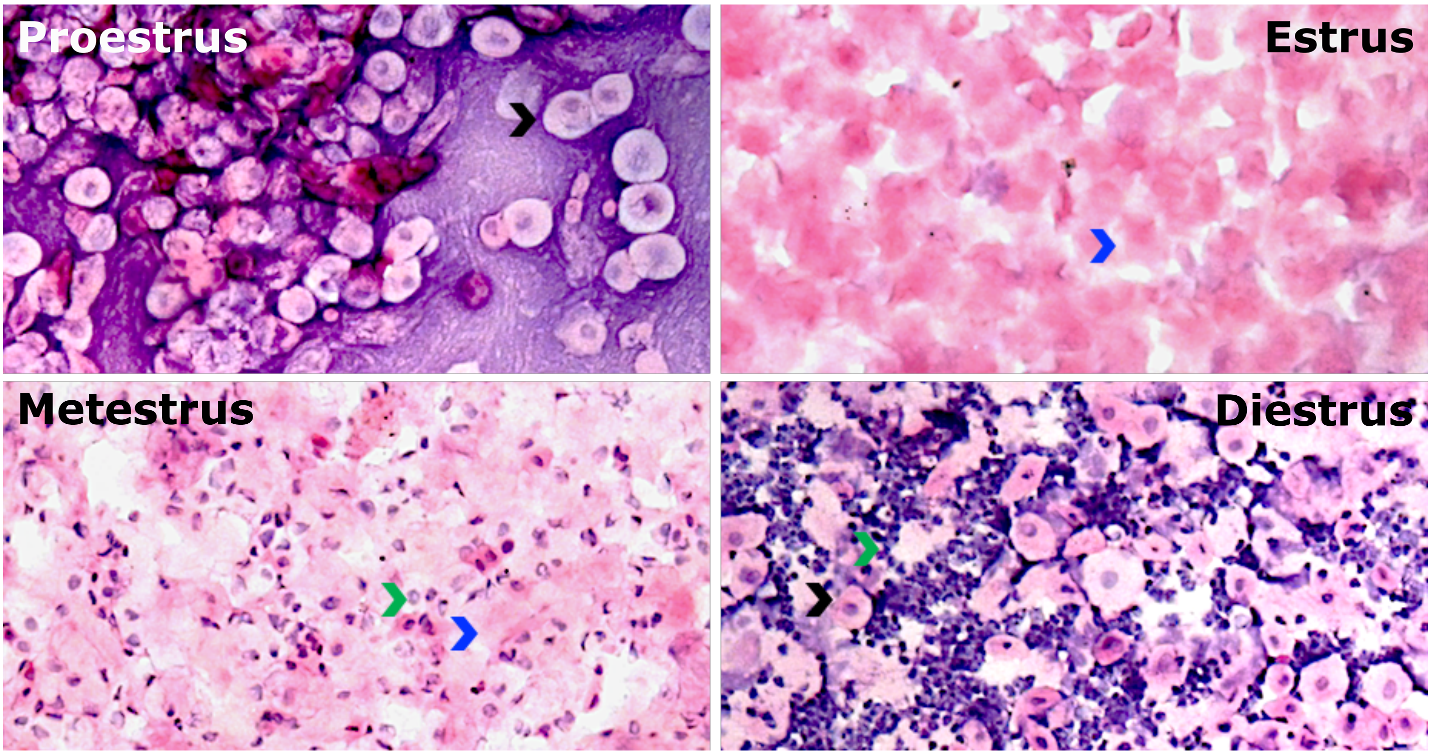

Suppl. Figure 6: Identification of mouse estrus cycle.
Cell types contained in the vaginal lavage were assessed under a light microscope in order to ascertain the estrus cycle stage which was characterized by the presence or absence of leukocytes and nucleated epithelial or cornified epithelial cells. Shown here are lavage samples that were subsequently air-dried and stained with hematoxilin and eosin. Proestrus shows a predominance of nucleated epithelial cells (black arrow head), while estrus consists mainly of cornified epithelial cells (blue arrow head). Metestrus and diestrus both show a predominance of leukocytes (green arrow head) and can be additionally differentiated by the presence of cornified and nucleated epithelial cells in metestrus and diestrus respectively (Caligioni, 2009; Byers et al. 2012). Representative pictures taken under 10x magnification on a light microscope.

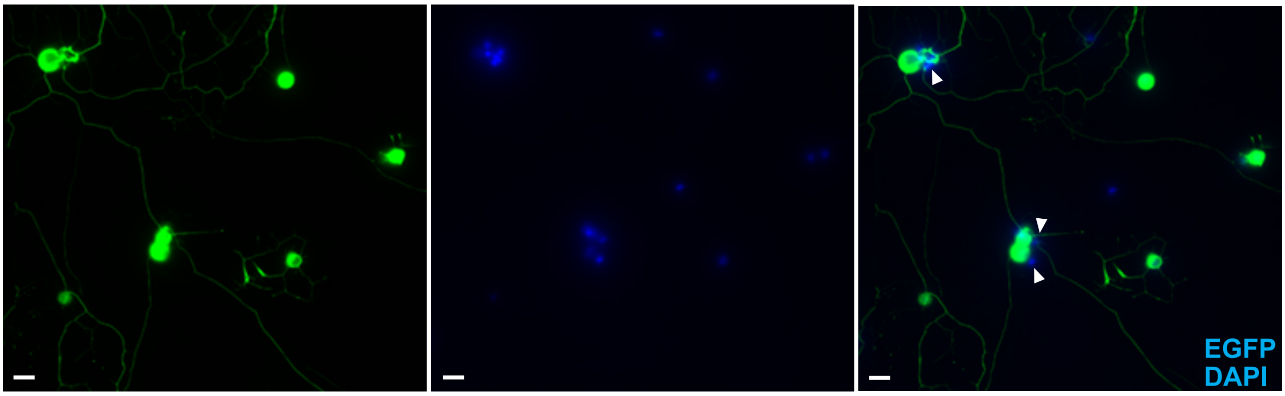

Suppl. Figure 7: Cultured DRG neurons after flourescence-activated cell sorting (FACS).

Primary neurons derived from Advillin-EGFP mice were culture for 48h after being sorted. On average,around 50% of neurons presented with satellite cells attached to them (nuclei marked by white arrows). Scale bars: 20 μm.
